# Glycogen synthase kinase 3α and 3β have distinct functions during cardiogenesis of zebrafish embryo

**DOI:** 10.1186/1471-213X-7-93

**Published:** 2007-08-03

**Authors:** Huang-Chieh Lee, Jen-Ning Tsai, Pei-Yin Liao, Wei-Yuan Tsai, Kai-Yen Lin, Chung-Cheng Chuang, Chi-Kuang Sun, Wen-Chang Chang, Huai-Jen Tsai

**Affiliations:** 1 Institute of Molecular and Cellular Biology, National Taiwan University, NO. 1, Roosevelt Road, Sec. 4, Taipei 106, Taiwan; 2 School of Medical Laboratory and Biotechnology, Chung Shan Medical University, Taichung 402, Taiwan; 3 Graduate Institute of Photonics and Optoelectronics and Department of Electrical Engineering, National Taiwan University and Research Center for Applied Sciences, Academia Sinica, Taipei 10617, Taiwan; 4 Institute of Biochemical Sciences, National Taiwan University, Taipei 106, Taiwan; 5 Institute of Biological Chemistry, Academia Sinica, Nankang 115, Taiwan

## Abstract

**Background:**

Glycogen synthase kinase 3 (GSK3) encodes a serine/threonine protein kinase, is known to play roles in many biological processes. Two closely related GSK3 isoforms encoded by distinct genes: GSK3α (51 kDa) and GSK3β (47 kDa). In previously studies, most GSK3 inhibitors are not only inhibiting GSK3, but are also affecting many other kinases. In addition, because of highly similarity in amino acid sequence between GSK3α and GSK3β, making it difficult to identify an inhibitor that can be selective against GSK3α or GSK3β. Thus, it is relatively difficult to address the functions of GSK3 isoforms during embryogenesis. At this study, we attempt to specifically inhibit either GSK3α or GSK3β and uncover the isoform-specific roles that GSK3 plays during cardiogenesis.

**Results:**

We blocked *gsk3α *and *gsk3β *translations by injection of morpholino antisense oligonucleotides (MO). Both *gsk3α*- and *gsk3β*-MO-injected embryos displayed similar morphological defects, with a thin, string-like shaped heart and pericardial edema at 72 hours post-fertilization. However, when detailed analysis of the *gsk3α*- and *gsk3β*-MO-induced heart defects, we found that the reduced number of cardiomyocytes in *gsk3α *morphants during the heart-ring stage was due to apoptosis. On the contrary, *gsk3β *morphants did not exhibit significant apoptosis in the cardiomyocytes, and the heart developed normally during the heart-ring stage. Later, however, the heart positioning was severely disrupted in *gsk3β *morphants. *bmp4 *expression in *gsk3β *morphants was up-regulated and disrupted the asymmetry pattern in the heart. The cardiac valve defects in *gsk3β *morphants were similar to those observed in *axin1 *and *apc*^*mcr *^mutants, suggesting that GSK3β might play a role in cardiac valve development through the Wnt/β-catenin pathway. Finally, the phenotypes of *gsk3α *mutant embryos cannot be rescued by *gsk3β *mRNA, and vice versa, demonstrating that GSK3α and GSK3β are not functionally redundant.

**Conclusion:**

We conclude that (1) GSK3α, but not GSK3β, is necessary in cardiomyocyte survival; (2) the GSK3β plays important roles in modulating the left-right asymmetry and affecting heart positioning; and (3) GSK3α and GSK3β play distinct roles during zebrafish cardiogenesis.

## Background

Glycogen synthase kinase 3 (GSK3) encodes a multifunctional serine/threonine protein kinase, which is ubiquitously expressed in organisms ranging from yeasts to mammals [1-3]. GSK3 is, therefore, very important in the cellular signaling network. In addition to playing pivotal roles in the canonical Wnt and PI3K-PKB/AKT pathways, it has been shown to phosphorylate glycogen synthase, eLF2B, NFAT, c-jun, CyclinD1, NF-kB, as well as many others [4]. GSK3 is involved in many biological processes, including cell survival, tumorigenesis, and developmental patterning.

There are two closely related GSK3 isoforms encoded by distinct genes: GSK3α (51 kDa) and GSK3β (47 kDa) [5]. The difference in size is due to a glycine-rich extension at the N-terminus of GSK3α. GSK3α and GSK3β are highly homologous within their kinase domains [6]. Homologues of GSK3 isoforms from species as distant from each other as flies, zebrafishes and humans display over 90% sequence similarity within the kinase domain [7,8].

Despite that GSK3α and GSK3β share common substrates, their expression patterns, substrate preferences, regulation, and cellular functions are not identical [1,6,9,10]. *In vitro *study reveals that GSK3α and GSK3β are inactivated by phosphorylation of a specific N-terminal serine residue (Ser-21 in GSK3α; Ser-9 in GSK3β) catalyzed by either MAPKAP kinase-1/or p70^S6K ^[11,12], whereas protein kinase C phosphorylates and partially inhibits GSK3β, but not GSK3α [13]. In humans, only GSK3α is deactivated by insulin during physiological conditions [14,15], whereas supraphysiological insulin injection in the rat leads to deactivation of both GSK3α and GSK3β [15,16]. Although differential regulations by the two isoforms of GSK3 were proposed, the exact roles of GSK3α and GSK3β and endogenous targets of such regulation remain to be investigated.

Several groups have identified small-molecule GSK3 inhibitors [17,18]. Most drugs bind to the ATP pocket of GSK3 and compete with ATP. However, these inhibitors are not only inhibiting GSK3, but are also affecting CDK kinase (2 and 5) and many other kinases. In addition, there appears to be only a single amino acid difference (Glu196 in GSK3α, Asp133 in GSK3β), making it difficult to identify an inhibitor that can be selective against GSK3α or GSK3β [19]. This finding is why it is difficult to analyze the exact roles of GSK3α and GSK3β *in vitro *and *in vivo*.

Recent years, numerous studies indicate that GSK3 negatively regulates cardiac hypertrophy [20-22]. Despite that GSK3β functions as a negative regulator of cardiac hypertrophy, GSK3 also plays an important role in regulating cardiac development. Transgenic mice over-expressing GSK3β in the heart have impairments of postnatal cardiomyocyte growth and abnormal cardiac contractile function [23]. In *Xenopus*, injection of *gsk3β *mRNA in embryos induces expression of Nkx2.5 and Tbx5 [24]. Oral treatment with lithium, a mood-stabilizing drug that is inhibitory for GSK3, in pregnant women showed a higher incidence of congenital heart defects in babies [25-27]. These findings prove that GSK3 might be involved in heart development. Unfortunately, disruption of the *gsk3β *gene in mice results in embryonic lethality caused by severe liver degeneration [9], and no report is available to demonstrate that cardiac defects are happened in GSK3β mutants. Thus, whether the roles of GSK3α and GSK3β in different species are conserved remain to be investigated. Moreover, the roles of GSK3 in cardiac development are still unclear. Also, whether GSK3α and GSK3β play similar roles in heart development is ambiguous.

We have previously identified two zebrafish homologues related to mammalian GSK3: zebrafish GSK3α and GSK3β [8]. In this report, we have taken advantage of the zebrafish system to address the distinct roles of GSK3α and GSK3β during heart development of zebrafish. Our findings suggest that, in zebrafish, GSK3α, but not GSK3β, is necessary in cardiomyocyte survival; whereas the GSK3β isoform plays important roles during zebrafish cardiogenesis, modulating the left-right asymmetry and affecting heart positioning.

## Results

### Knockdown of *gsk3α *and *gsk3β *display similar heart defects in the embryos

**Figure 1:**
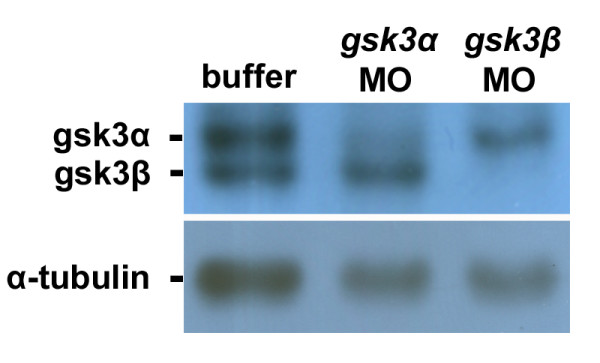
**Injection of translation inhibitors *gsk3α*- and *gsk3β *-MO into embryos can specifically reduce the protein levels of GSK3α and GSK3β, respectively**. The total protein lysate extracted from seven zebrafish embryos at 24 hpf was loaded on each lane and analyzed by western blot. The antibody used is indicated in the left of each blot. Anti-GSK3 antibody enables to recognize both GSK3α and GSK3β proteins; anti-α-tubulin antibody was used as a loading control. The protein levels of GSK3α and GSK3β were reduced greatly in the protein lysates extracted from the *gsk3α *– and *gsk3β *-MO-injected embryos, respectively.

To address the role of GSK3 during zebrafish cardiogenesis, we designed *gsk3α*- and *gsk3β*-MO for specifically inhibiting the translation of *gsk3α *and *gsk3β*, respectively. When the protein lysate was extracted from *gsk3α *– and *gsk3β*-MO-injected embryos at 24 hours postfertilization (hpf), Western blot analysis was performed by using isoform-specific antibodies. Results showed that the protein levels of GSK3α and GSK3β were largely reduced in the protein extracts from *gsk3α *– and *gsk3β *-morphants, respectively (Fig. 1), suggesting that the MOs we designed in this study were isoform-specific.

**Figure 2:**
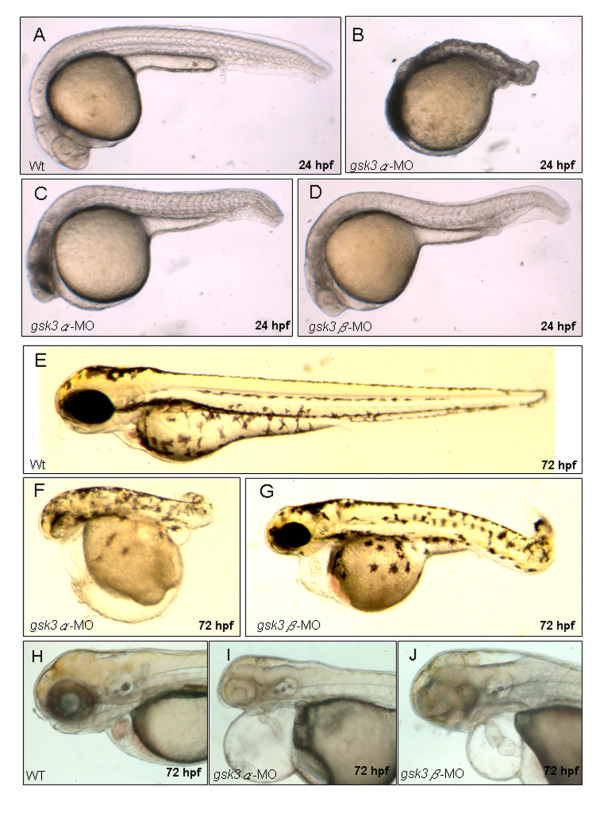
**The morphological defects in *gsk3α *and *gsk3β *morphants**. Wild-type embryos (A, E, H), *gsk3α *(B, C, F, I), and *gsk3β *(D, G, J) morphants. The 24 hpf *gsk3α *morphants have mild (C) to severe (B) defects in axis formation. At 72 hpf, both *gsk3α *and *gsk3β *morphants displayed pericardial edema (F, G, I, J) and an unlooped, stretched heart (I, J).

Similar morphological defects of the heart were observed in *gsk3α*- and *gsk3β*-MO-injected zebrafish embryos at 72 hpf, such as a thin and string-like shape, pericardial edema, and blood pooling (Fig. 2F, G, I, J). These defects occurred initially in some 2 days postfertilization (dpf) morphants, and then were predominantly observed in most 3- and 4-dpf morphants. Although the heart defects were similar between *gsk3α*- and *gsk3β*-MO-injected zebrafish embryos, the defects of the *gsk3α *morphants were more severe than those of the *gsk3β *morphants (Fig. 2B, C, F vs. 2D, G). Around 40% of the *gsk3α *morphant defects were lethal due to an absent body axis during 24 hpf (Fig. 2B), and the remainder of the surviving *gsk3α *morphants suffered from an incomplete formation of axis (Fig. 2C), suggesting that *gsk3α *and *gsk3β *may function differently during cardiogenesis, although they cause similar heart defects. We also noticed that the percentage of heart abnormalities was dependent on the concentration of the injected *gsk3α*- and *gsk3β*-MO (Table 1). When 0.5 ng of *gsk3α*-MO was injected into 1-celled stage embryos, we found that 41.8% (n = 146) of embryos displayed a string-like-shape heart; whereas when 2 ng *gsk3α*-MO were injected, 88.2% (n = 212) of embryos appeared similar heart defect. Similarly, 2 ng *gsk3β*-MO caused 30.2% (n = 126) of embryos to suffer a string-like-shape heart at 72 hpf; whereas 6 ng *gsk3β*-MO caused 87.5% (n = 288) of embryos to have similar heart defect. These results indicated that the effect of *gsk3*-MO on embryogenesis was dosage-dependent and specific.

**Table 1:** Morphological phenotypes of zebrafish embryos derived from fertilized eggs injected with different materials

Injected materials	Concentration	Number of embryos surviving among number of injected eggs	Wild-type phenotype	Abnormal Heart Development
*gsk3α *-MO	0.5 ng	146/155 (94.2%)	85 (58.2%)	61 (41.8%)
*gsk3α *-MO	1 ng	176/191 (92.1%)	61 (34.6%)	115 (65.4%)
*gsk3α *-MO	2 ng	212/273 (77.7%)	25 (11.8%)	187 (88.2%)
*gsk3β *-MO	2 ng	126/129 (97.7%)	88 (69.8%)	38 (30.2%)
*gsk3β *-MO	4 ng	137/144 (95.1%)	69 (50.4%)	68 (49.6%)
*gsk3β *-MO	6 ng	288/314 (91.7%)	36 (12.5%)	252 (87.5%)
*axin1*-MO	6 ng	151/164 (92.1%)	27 (17.9%)	124 (82.1%)
*dsRed *mRNA	100 pg	68/68 (98.8%)	83 (100%)	0 (0%)
Control MO	2 ng	105/108 (97.2%)	101 (96.2%)	4 (3.8%)
Control MO	6 ng	112/125 (89.6%)	103 (93.6%)	7 (6.4%)
*gsk3α *-mRNA_*gsk3α *-MO	50 pg + 2 ng	59/69 (85.5%)	18 (30.5%)	41 (69.5%)
*gsk3α *-mRNA_*gsk3α *-MO	100 pg + 2 ng	93/118 (78.8%)	51 (54.8%)	42 (45.2%)
*gsk3β *-mRNA_*gsk3α *-MO	50 pg + 2 ng	72/90 (80.0%)	9 (12.5%)	63 (87.5%)
*gsk3β *-mRNA_*gsk3α *-MO	100 pg + 2 ng	91/134 (67.2%)	5 (5.5%)	86 (94.5%)
*gsk3β *-mRNA_*gsk3β *-MO	50 pg + 6 ng	73/77 (94.8%)	45 (61.6%)	28 (38.4%)
*gsk3β *-mRNA_*gsk3β *-MO	100 pg + 6 ng	128/144 (89.0%)	96 (75.0%)	32 (25%)
*gsk3α *-mRNA_*gsk3β *-MO	50 pg + 6 ng	83/109 (76.1%)	15 (18.1%)	68 (81.9%)
*gsk3α *-mRNA_*gsk3β *-MO	100 pg + 6 ng	92/127 (72.4%)	8 (8.7%)	84 (91.3%)
*dsRed*-mRNA_*gsk3α *-MO	100 pg + 2 ng	83/96 (86.5%)	7 (8.4%)	76 (91.6%)
*dsRed*-mRNA_*gsk3β *-MO	100 pg + 6 ng	75/82 (91.4%)	13 (17.3%)	62 (82.7%)

Fertilized eggs were injected at the 1-cell stage, and then *gsk3α *morphants were observed at 36 to 48 hpf; *gsk3β *morphants were observed the heart positioning at 24 to 36 hpf. Results are from three independent experiments. *dsRed *mRNA: served as a negative control.

### Heart defects caused by *gsk3α*- and *gsk3β*-MO are induced differently

We investigated whether the MO-induced defects could be rescued by co-injecting synthetic *gsk3α *or *gsk3β*-mRNA with its corresponding MOs, and vice versa. Results showed that co-injection of *gsk3α*-MO with synthetic *gsk3α*-mRNA could effectively rescue the defects caused by the injection of *gsk3α*-MO alone (Table 1). Similarly, the *gsk3β*-MO-induced defects were rescued by injection of *gsk3β*-mRNA. However, the synthetic *gsk3α*-mRNA did not rescue the *gsk3β*-MO-induced phenotype, and vice versa (Table 1). This evidence clearly demonstrates that two isoforms of GSK3 are necessary for heart development, but the function of GSK3α and GSK3β is not redundant, suggesting that GSK3α and GSK3β play specific roles in cardiogenesis during zebrafish development.

**Figure 3:**
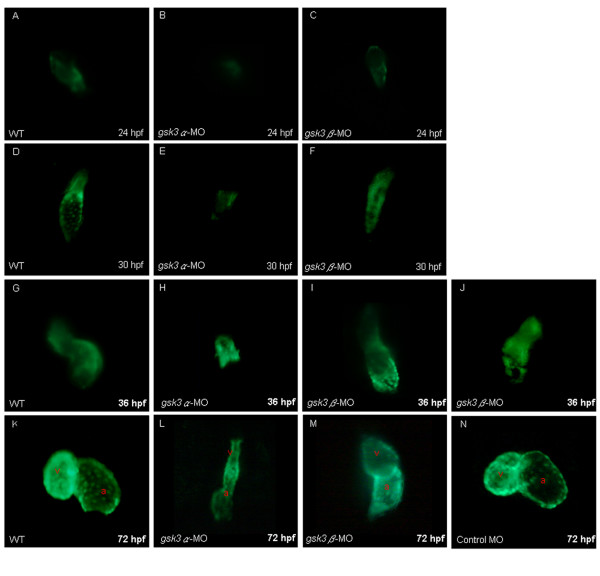
**The cardiac defects induced by the knockdown of zebrafish GSK3α and GSK3β**. Anti-sense morpholino oligonucleotide (MO), which was designed to specifically inhibit the translation of either *gsk3α*-(*gsk3α*-MO) or *gsk3β*-mRNA (*gsk3β*-MO), was injected into one-celled stage embryos and the heart morphology was observed at the stage as indicated. The elongation of heart tube was normally developed at 24 hpf in the wild-type (A) and in the *gsk3β *morphants (C); whereas the heart of *gsk3α *morphant did not elongate to from a heart-tube (B). The wild-type (D) and *gsk3β *morphant's heart (F) developed normally at 30 hpf, but the heart of *gsk3α *morphant was still retardant development at 30 hpf (E), and even ceased at heart-cone stage at 36 hpf (F). Compared to the wild-type (G), however, the heart positioning was abnormally in the *gsk3β *morphant at 36 hpf (I, J). Eventually, both *gsk3α *and *gsk3β *morphants displayed an unlooped and stretched heart (L, M). The heart morphology of embryos injected with the control MO was also observed at 72 hpf (N). a: atrium; v: ventricle.

We injected either *gsk3α*- or *gsk3β*-MO into embryos derived from the transgenic line Tg(*cmlc2: gfp*), in which the GFP is expressed specifically in heart, resulting in a good material to monitor cardiac development of zebrafish [29]. In the wild-type embryos, the heart precursor cells completed their *in situ *formation, elongated, and jogged to the left at 24 hpf; started looping at 30 hpf; and completed looping at 48 hpf [34]. However, we observed that heart development was retarded, failing to elongate at 24 hpf (Fig. 3B) and even ceasing at heart-cone stage without further morphogenesis to a heart tube at [30-36] hpf (Figs. 3E, H) in the *gsk3α*-MO-injected embryos. We observed defective hearts as stretched to a thin and string-like shape at 72 hpf (Fig. 3L). Nevertheless, unlike in *gsk3α *morphants, elongation of the heart tube in *gsk3β *morphants at 24 hpf was as normal as in wild-type zebrafish (Fig. 3C), but heart looping was incomplete from 30 to 36 hpf (Figs. 3F, I), resulting in a stretched heart at 72 hpf (Fig. 3M).

In addition, we have designed to an experiment for using a standard negative control morpolino (MO) injection: 5'-CCTCTTACCTCAGTTACAATTTATA-3' (Gene Tools, USA). This oligo has no target, no significant biological activity. After 2 and 6 ng of this control MO were injected, no any defects were observed at 24 hpf. The morphology and development of heart appeared normally (see Additional file 1 and Figure 3N). These results reveal that the defects induced by the *gsk3α*- and *gsk3β*-MO are specific in this study.

### Effect of GSK3 on the number of cardiomyocytes is isoform-specific

**Figure 4:**
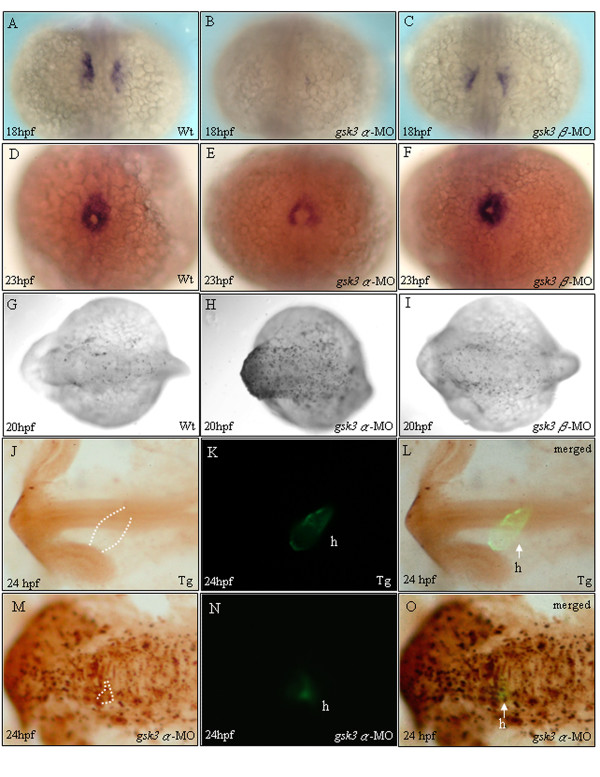
**The heart defects in *gsk3α *morphants weredue to a reduced cardiomyocyte population size**. Dorsal (A-I) and lateral (J-O) views of embryos stained by *in situ *hybridization (A-F) and TUNEL assay (G-L). Whole mount *in situ *hybridization staining with *cmlc2 *at 18 and 23 hpf received that *gsk3α*-MO causes a repressive influence on cardiomyocyte formation (B, E). The heart defect in *gsk3α *morphants was due to the reduction of cardiomyocyte population size. However, *gsk3β *morphants display normal cardiomyocyte formation (C, F) compared to wild-type embryos (A, D). TUNEL labeling was evident throughout the head of *gsk3a*-MO-injected embryos (H), especially in the head, but was limited in the head of controls (G) and *gsk3β *morphants (I). Compared to embryos derived from the transgenic line, Tg(*cmlc*2:EGFP), which has heart-specific GFP (K), we observed that the heart of *gsk3α *morphant did not elongate to form a heart-tube and the GFP signal was very faint at 24 hpf (N). Panels L and O are the merged images from J and K, and M and N, respectively. The apoptotic signals were co-localized with the heart-specific GFP signal, indicating that the reduced cardiomyocyte numbers was due to apoptosis in heart (O). h: heart.

Compared to that of wild-type and *gsk3β *morphants, the GFP signals in cardiomyocytes of *gsk3α *morphants were greatly reduced (Fig. 3B). To investigate whether the reduced GSK3α level affects the cardiomyocyte number, we used a cardiomyocyte marker, cardiac myosin light chain 2 (*cmlc2*), to detect cells at heart-field and heart-cone stages. We found that the number of *cmlc2*-positive cells was greatly reduced in *gsk3α *morphants at both heart-field and heart-cone stages (Fig. 4B, E), indicating that the cardiomyocyte number was greatly reduced in the *gsk3α *morphants. These results suggest that the retarded heart development in *gsk3α *morphants is due to the decreased number of cardiomyocytes during early cardiogenesis. In contrast, *gsk3β *morphants displayed normal *cmlc2 *staining (Fig. 4C, F), indicating that cardiomyocyte number remains unchanged in *gsk3β *morphants. These results also clearly demonstrate that GSK3α and GSK3β play distinct roles during cardiogenesis.

### Apoptosis occurs in the head of *gsk3α *knockdown embryos

The pronounced degeneration in the head of *gsk3*α morphants at [18-30] hpf were also observed (Fig. 2C). To confirm whether the reduced cardiomyocyte number in *gsk3α *morphants was due to apoptosis, the embryos were analyzed by Terminal deoxynucleotidyl Transferase Biotin-dUTP Nick End Labeling (TUNEL) assay after MO injection. In wild-type embryos at 20 hpf, apoptosis was low (Fig. 4G). However, in *gsk3α *morphants at the same stage, apoptosis was pronounced throughout the axis, especially in the head (Fig. 4H) but was limited in the head of controls (G) and *gsk3β *morphants (I). Moreover, In *gsk3a*-morphants, the GFP signal was very faint at 24 hpf (Fig. 4N). The apoptotic signals were co-localized with the heart-specific GFP signal, indicating that the reduced cardiomyocyte numbers was due to apoptosis in heart (Fig. 4O). Taken together, the heart defects in *gsk3α *morphants was due to the reduced number of cardiomyocytes, which results from apoptosis in the head.

### GSK3β, but not GSK3α, is involved in the cardiac positioning

**Figure 5:**
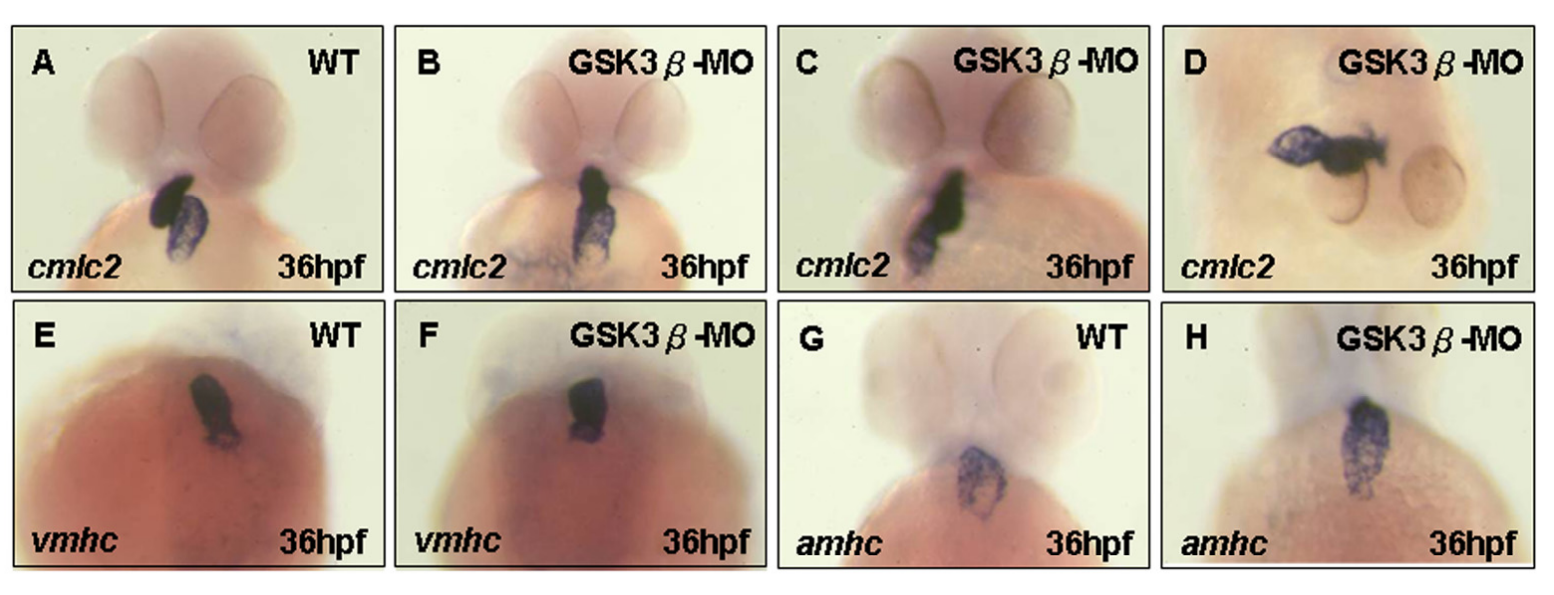
**Cardiac positioning is *gsk3β*-dependent, but the chamber-specific patterning is not**. A-C, E-H) ventral view, (D) dorsal view of wild-type (A, E, G), and *gskβ *morphants (B-D, F, H) at 36 hpf. *in situ *hybridization with *cmlc2 *staining revealed that randomized looping was observed in *gsk3β *morphants (B-D). The expression of *vmhc *(F) and *amhc *(H) appeared normal in *gsk3β *morphants.

Although the heart of *gsk3β*-MO-injected embryos eventually becomes a string-like shape, we found that the cardiomyocyte development was not affected in the *gsk3β *morphants during early cardiac development, suggesting that GSK3β may play a unique role in cardiac morphogenesis. Whole-mount *in situ *hybridization of the *cmlc2 *probe at 36 hpf, outlining cardiac looping, was marked by a rightward bending in the ventricle in wild-type embryos (Fig. 5A). However, no looping was observed in *gsk3β *morphants (Fig. 5B–D). Upon detailed analysis of the early (jogging) and late (looping) stages of cardiac positioning in the *gsk3β *morphant heart (Table 2), we found that heart positioning was severely disrupted in *gsk3β *morphants and that the extent of the defect was proportional to the amount of *gsk3β*-MO we injected. The majority of *gsk3β *morphant hearts failed to jog (69.9%; 65/93). Moreover, this defect was frequently accompanied by no looping (45.3%) or L-looping (14.4%) of the heart tube, compared to wild-type, which has correct left-jogging (93.1%; 67/72) and D-looping (92.4%; 122/132). These results indicate that knockdown of GSK3β resulted in a severe disruption of jogging and looping of cardiac positioning. However, we found that the ventricle-specific marker *vmhc *and the atrium-specific marker *amhc *were normally transcribed in *gsk3β *morphants (Fig. 5E, G vs. 5F, H), suggesting that GSK3β might not affect the chamber-specific pattern of gene expression, although normal heart looping was not completed. We also noted that the heart positioning in *gsk3α *morphants was delayed but that correct jogging (left-jog) and looping (D-loop) were observed at [36-48] hpf, indicating that GSK3α was not involved in heart positioning.

**Table 2:** Knockdown GSK3β levels that disrupt normal cardiac jogging and looping

	No. of embryos	Left-jog	No-jog	Right-jog	No. of embryos	D-loop	No-loop	L-loop
Uninjected	72	93%	2.8%	4.2%	132	92.5%	3%	4.5%
2 ng *gsk3α *-MO	63	87.3%	7.9%	4.8%	74	93.2%	4.1%	2.7%
1 ng *gsk3α *-MO	58	93.1%	5.2%	1.7%	90	94.5%	2.2%	3.3%
6 ng *gsk3β *-MO	93	17.2%	69.9%	12.95	159	40.3%	45.3%	14.4%
4 ng *gsk3β *-MO	86	22.1%	67.4%	10.5%	101	40.6%	44.6%	14.8%
2 ng *gsk3β *-MO	62	45%	50%	5%	107	70.1%	22.4%	7.5%

*gsk3α *morphants were analyzed for heart positioning at 36 to 48 hpf; gsk3β morphants were analyzed for heart positioning at 24 to 36 hpf.

### GSK3β mediates *bmp4 *and *lefty-1 *in cardiac positioning and is required for left-right patterning

**Figure 6:**
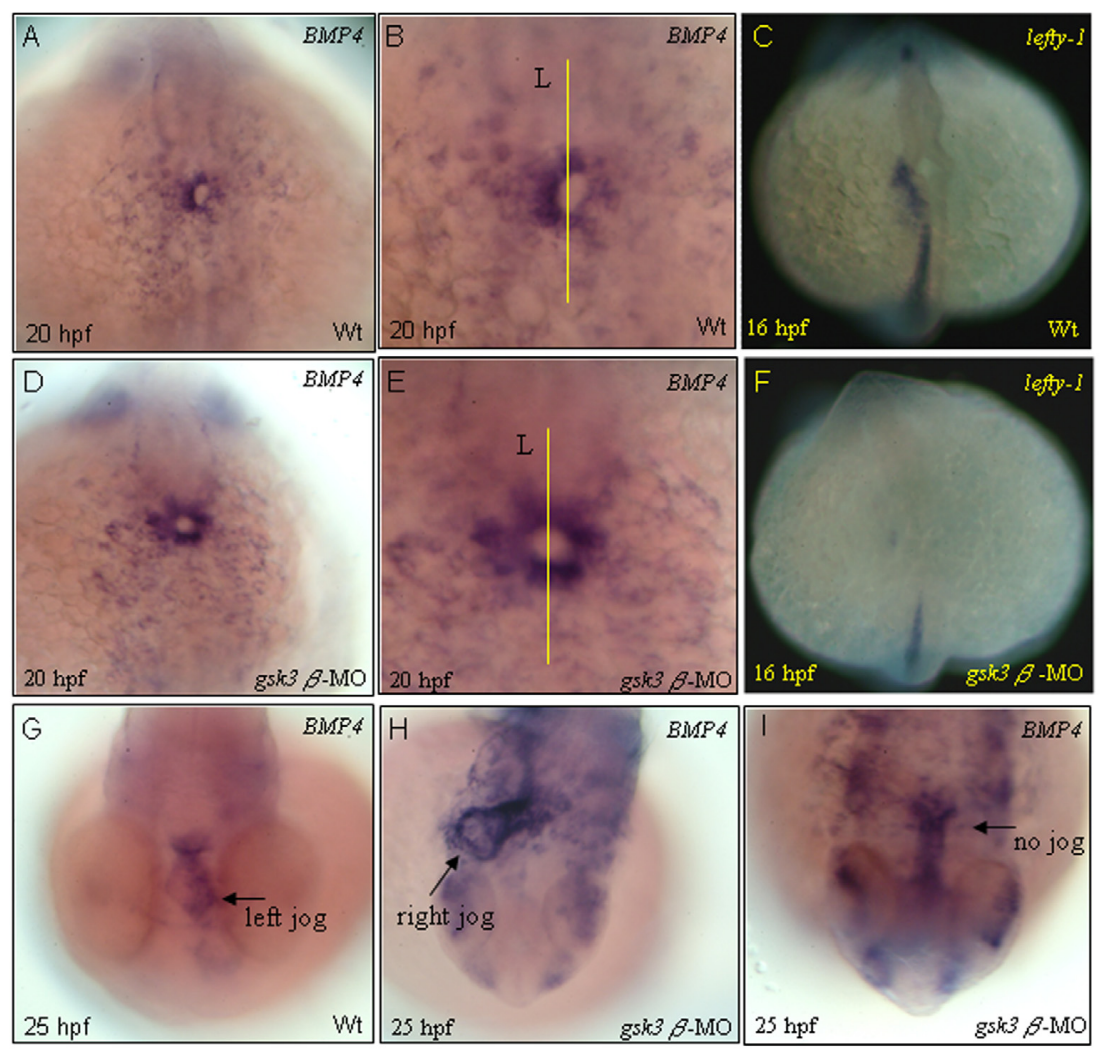
**Heart asymmetry was affected in *gsk3β *morphants**. Normally, *bmp4 *transcripts accumulate predominantly on the left side of the heart tube at 20 hpf (A, B), and the left-predominant *bmp4 *asymmetry persists through the stages of jogging (G). However, in *gsk3β *morphants, the expression of *bmp4 *becomes symmetrical at 20 hpf (B, D). In *gsk3β *morphants, in which the heart fails to jog, *bmp4 *is more evenly distributed in the heart region (H, I). The left-sided *lefty-1 *domain was greatly reduced in *gsk3β *morphant hearts at 16 hpf (F). All are dorsal views. B, E are higher magnifications of A, D, respectively. Lines mark the midline. L, embryo left.

Cardiac *bmp4 *is an integral component involved in the asymmetric signaling pathway and interprets left-right information for the zebrafish embryo heart [35]. The *bmp4 *transcripts became markedly asymmetric, with far more on the left side than on the right side of the heart ring at 20 hpf (Fig. 6A, B), just before jogging. This left-predominant asymmetry persists through the stages of jogging (25 hpf, Fig. 6G). However, the pattern of *bmp4 *expression in *gsk3β *morphants was symmetrical before jogging (Fig. 6D, E) and ectopic around the heart-tube stage at 25 hpf, thereby disrupting the pattern of left-predominant asymmetry (Fig. 6H, I). Moreover, another asymmetric marker, *lefty-1 *[36], lost its expression domain in the left side of the midline in *gsk3β *morphants (Fig. 6C, F). We propose that GSK3β mediates *bmp4 *and *lefty-1 *in cardiac positioning.

### GSK3β modulates valve formation and heart position through Wnt/β-catenin signaling

**Figure 7:**
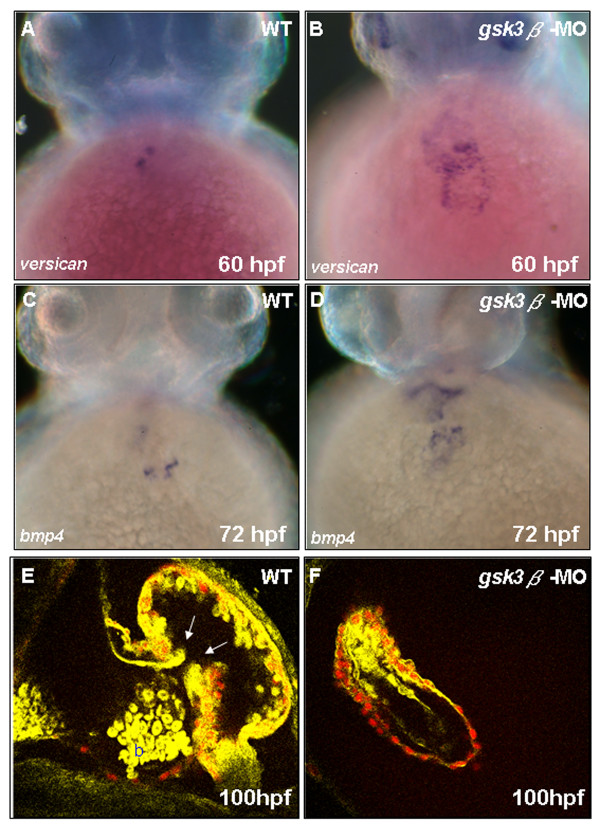
**GSK3β modulates zebrafish cardiac valve formation**. Whole-mount *in situ *hybridization with *bmp4 *and *versican *staining reveals that cardiac valve development was affected in *gsk3β *morphants. At 60–72 hpf,*versican *(A, B) and *bmp4 *(C, D) expression was greatly up-regulated in *gsk3β *morphants. Tg(*cmlc2: Hc-RFP*) embryos were injected with *gsk3β*-MO and observed by *in vivo *two-photon fluorescence imaging of a live transgenic zebrafish heart at 100 hpf. The endocardial cells and blood are labeled yellow; the Hc-GFP-positive myocardial cells are labeled red. Valves are clearly observed in wild-type embryos (E; white arrows), but not in *gsk3β *morphants (F). b, blood cells; V, ventricle; A, atrium.

Many morphological defects of heart were found in the *gsk3β*-MO-injected zebrafish embryos. Moreover, when we used the valve markers *bmp4 *and *versican *to detect the *gsk3β*-MO-injected embryos at 60–72 hpf, we found that these valve markers were markedly up-regulated in the heart (Fig. 7A–D), suggesting that GSK3β might also be involved in the formation of cardiac valves. Thus, we used a two-photon fluorescence image to directly observe the valve formation of embryos derived from the transgenic zebrafish line Tg(*cmlc2:Hc-RFP*; 28). The yellow color shown in our nonlinear microscopy image (valves and red blood cells) is corresponding to the image modality taken by the Third-Harmonic-Generation Microscopy. Valves were normally formed in the wild-type embryos (Fig. 7E), but valves of embryos injected with *gsk3β*-MO were totally absent (Fig. 7F).

Hurlstone et al [37] reported that cardiac valve formation is severely affected in zebrafish APC mutants *(apc*^*mcr*^*)*. Furthermore, when *axin1*, another key component in the Wnt pathway, is knocked down, either a reduction or absence of heart positioning of the heart tube was frequently observed (see Additional file 2A–D). GSK3 is known to be important in the canonical Wnt pathway, and the defective valves and hearts in *gsk3β*-MO-injected embryos were identical to those observed in the *apc*^*mcr *^mutants and *axin1 *morphants, suggesting that GSK3β modulates cardiac development through Wnt/β-catenin signaling.

## Discussion

GSK3β knockout mice display an embryonic lethal phenotype, indicating that GSK3α does not compensate for the loss of GSK3β [9]. Furthermore, the activity of GSK3α, but not GSK3β, is required for the production of amyloid-β in the brain of patients with Alzheimer's disease [38]. All these findings indicate that there may be isoform-specific functions of GSK3, although they exhibit similar biochemical and substrate properties *in vitro *[7]. However, to clearly delineate the biological functions of these two GSK3 isoforms *in vivo *is difficult and little reported. In this report, we study the roles of GSK3α and GSK3β in the cardiogenesis of zebrafish using a loss-of-function approach. The loss of either GSK3α or GSK3β results in abnormal development of heart. Yet, the role that GSK3 plays in cardiogenesis is in an isoform-specific manner. GSK3α plays a role in the survival of cardiomyocytes, whereas GSK3β plays a role in heart left-right biased positioning, modulated through the Wnt/β-catenin signaling pathway.

### GSK3α and GSK3β play different roles during zebrafish embryogenesis

Although, knockdown of *gsk3α *and *gsk3β *causes similar defective phenotypes, such as an unlooped and stretched heart, pericardial edema, blood pooling. We used *gsk3α*-MO and *gsk3β*-MO in the transgenic zebrafish line *Tg*(*cmlc2:GFP*), in which GFP is expressed in the myocardium specifically, to modulate and observe, in real-time, the different defective phenotypes. The hearts of *gsk3α *morphants failed to elongate at 24 hpf. We prove that the heart defects induced by the *gsk3α*-MO are due to a decreased number of cardiomyocytes. On the other hand, the *gsk3β*-MO-injected embryos develop normally before the onset of cardiac jogging. Defective heart positioning is observed after 26 hpf. Rescue experiments revealed that GSK *3α *and GSK*3β *do not function redundantly. Taken together, we conclude that each isoform of GSK3 plays its own distinct role during cardiogenesis of zebrafish.

### GSK3α, but not GSK3β, is involved in apoptosis during early embryogenesis

GSK3 plays an important role in the regulation of apoptosis/cell survival through the activation of caspase3 [39,41,42]. These findings support a role of GSK3β in controlling apoptosis. Many studies reporting the affect of GSK3β on apoptosis have been confirmed by using GSK3 inhibitors, including lithium, the first known inhibitor, and many synthetic ones [43-45]. However, these inhibitors have many effects on cells and are not isoform-specific. Thus, whether GSK*3α *and GSK*3β *function redundantly or distinctly on cell survival is still ambiguous. In our study, extensive apoptosis is observed throughout the head region in the *gsk3α *morphants. On the other hand, only slight apoptosis is noticed in the *gsk3β *morphants, suggesting that GSK3α, but not GSK3β, is greatly involved in apoptosis during early embryogenesis. Moreover, embryos that are co-injected with *gsk3α*-MO and *gsk3β *mRNA do not show reduced apoptosis, suggesting that GSK*3α *and GSK*3β *do not function redundantly in cell survival.

### GSK3α-mediated apoptosis may not be implicated in Wnt signaling

The Wnt signaling are involved in cell proliferation and in apoptosis [46-49]. On the other hand, PKB/Akt, a major regulator of GSK3, also triggers a network that regulates cell cycle progression through inactivation of GSK3β [50]. It has been shown that PKB/Akt promotes cell survival in cardiac myocytes [51,52]. In zebrafish, *apc*^*mcr *^mutant's hearts are morphologically normal during early cardiogenesis, but they fail to undergo looping morphogenesis [37]. Both *apc*^*mcr *^and *axin1 *mutants *(mbl) *display cardiac defects that are similar to those of *gsk3β *morphants. However, no information is provided about apoptosis in *apc*^*mcr *^and *mbl *mutants. In this report, we find that apoptosis occurs in the head of *gsk3α *morphants. In addition, the *axin1*-MO-injected embryos and the *mbl *mutant of zebrafish have defects of looping morphogenesis in the heart, which are similar to defects occurring in the *gsk3β *morphants but are unlike defects occurring in the *gsk3α *morphants (see Additional file 2). Therefore, we know that GSK3α may not mediate apoptosis implicated in Wnt signaling because apoptotic signals do not increase in *axin1 *morphants (data not shown). It is worth studying which pathway is implicated in GSK3α-mediated apoptosis.

### *gsk3β *regulates *bmp4 *during cardiac development through Wnt/β-catenin signaling

The phenotypes of *apc*^*mcr *^and *mbl *mutants are similar to our results in that inhibition of GSK3β also causes unlooping heart tube, pericardial edema, and blood pooling [37]. In addition, valve development is totally lost in *gsk3β *morphants (Fig. 7), which is similar to that of *apc*^*mcr *^mutants. Ectopic expression of *bmp4 *in the heart at 24–72 hpf and ectopic expression of *versican *in the valve at 60–72 hpf are also observed in the *apc*^*mcr *^mutant and in the *gsk3β *morphant (Figs. 7A–D). Moreover, the retention of *bmp4 *symmetry is associated with disordered jogging, and we observe that *bmp4 *retention occurred in the *gsk3β *morphant. In addition, *bmp4 *is downstream of Wnt/β-catenin signaling in several systems [53,54]. Therefore, it is reasonable to conclude that GSK3β might regulate zebrafish cardiac development by means of the canonical Wnt/β-catenin signaling pathway.

### GSK3β may be involved in the regulation of T-box genes during cardiogenesis

Our study reveals that knockdown of *gsk3β *causes a string-like heart. This phenotype is similar to the *heartstrings *mutant, caused by mutation of the *tbx5 *[55]. Patients with Holt-Oram syndrome, one of the autosomal dominant human "heart-hand" disorders, are caused by mutations of *tbx5 *[56]. Both loss and gain of *tbx5 *functions result in an absence of heart looping and an alteration in cardiac-specific genes [57,58]. In our study, we demonstrate that *gsk3β *morphants appear to have multiple heart defects, such as a non-looping or reversed looping heart, slower heart rate, and no blood circulation (Figs. 3, 4). In addition, after we probe with fin markers, we prove that the pectoral fin of the GSK3β morphant fails to differentiate (see Additional file 3). In chick, Tbx5 and Tbx4 trigger limb initiation through activation of the Wnt/Fgf signaling cascade [59]. Therefore, we propose that GSK3β and Tbx5 might be involved in the same regulatory mechanism during cardiogenesis.

### Zebrafish is an alternative, promising model animal to study GSK3-specific inhibitors *in vivo*

GSK3 is a target of prominent drugs for treating many diseases, including Alzheimer's disease and diabetes mellitus. Substrate-competitive inhibitors, which compete for the substrate binding site of the kinase, are more likely to be highly specific inhibitors. Several ATP-competitive inhibitors of GSK3 have also been defined [17,18]. However, the development of new drug not only requires the identification of the target, but also requires validation in an *in vivo *system. Recently, Atilla-Gokcumen et al., [60] performed phenotypic experiments in zebrafish embryo which is served as an *in vivo *experiment to analyse the functions of novel GSK3 inhibitor, organometallic reagent (R)-7. In this study, we clearly distinguish the morphological defects in zebrafish GSK3α- and GSK3β-knockdown embryos. Therefore, these findings will surely provide new criteria for the *in vivo *validation of potential isoform-specific inhibitors of GSK3.

### Different biological function of GSK3 isoform in species

In this report, we have defined that GSK3α and GSK3β play distinct roles during zebrafish cardiogenesis. Moreover, the defective valves and hearts in *gsk3β*-MO-injected embryos were identical to those observed in the *apc*^*mcr *^mutants and *axin1 *morphants, suggesting that GSK3β modulates cardiac development through Wnt/β-catenin signaling. In addition, GSK3 is a critical regulator of Wnt signaling mechanism, several recent studies have shown that the components of the Wnt signaling play an important role in heart development [3]. However, heart defects are not reported in the GSK3β-knockout mice. One of reasons is that mice GSK3α might function redundantly to GSK3β during the heart development of mice. We also notice that the expression profiles of GSK3β in zebrafish and in *Xenopus *are different: zebrafish *gsk3β *is weakly detected until 50–60% epiboly, but *Xenopus gsk3β *is expressed strongly and constantly throughout embryogenesis [61,62]. Taken together, although GSK3 isoforms share highly conserved in their functional domain, the biological functions of GSK3 isoforms in different species are not identical.

## Conclusion

Our data indicate that GSK3α and GSK3β play distinct roles during zebrafish embryogenesis. GSK3α, but not GSK3β, is necessary in cardiomyocyte survival; GSK3β plays an important role in regulating left-right biased heart positioning during the cardiogenesis in zebrafish. We also demonstrate that the cardiac valve defects observed in *gsk3β *morphants were similar to those observed in *axin1 *and *apc*^*mcr*^mutants, suggesting that GSK3β modulates valve formation and heart position through Wnt/β-catenin signaling. Finally, the defects of *gsk3α *morphant embryos cannot be rescued by *gsk3β *mRNA, and vice versa, demonstrating that GSK3α and GSK3β are not functionally equivalent. Thus, we conclude that GSK3α and GSK3β play distinct roles during zebrafish cardiogenesis.

## Methods

### Zebrafish maintenance and observation

The zebrafish AB strain, transgenic lines Tg(*cmlc2:Hc-RFP*) and Tg(*cmlc2:GFP*) were raised and staged as previously described (28–30). The heart formation were observed under a fluorescent stereomicroscope MZ FLIII (Leica) and two-photon fluorescence microscope and Third-Harmonic-Generation Microscopy [28].

### Knockdown microinjection of zebrafish embryos

The following morpholino antisense oligonucleotides (MOs) were obtained from Gene Tools: *gsk3α*-MO, CCGCTGCCGCTCATTTCGGGTTGCA; *gsk3β*-MO, GTTCTGGGCCGACCGGACATTTTTC; *axin1*-MO, GCTAATGCGGTCATATCTCCTCTGC; standard negative control-MO, CCTCTTACCTCAGTTACAATTTATA. All MOs were prepared at a stock concentration of 1 mM and diluted to the desired concentration for microinjection into each embryo.

### Western blot

The embryos were dechorionated and deyolked with two extra washing steps as described in Link et al. [31]. Deyolked samples were dissolved in 2 μl of 2 × sodium dodecyl sulfate (SDS) sample buffer per embryo and incubated for 5 min at 95°C. After full-speed centrifugation for 1 min in a microcentrifuge to remove insoluble particles, samples were loaded on a 12% SDS gel (seven embryos per lane). Antibodies used were anti-GSK3 (Santa Cruz, SC-7291, 1:750) and anti-α-tubulin (Sigma-Aldrich, T9026, 1:750).

### Whole-mount *in situ *hybridization

Whole-mount *in situ *hybridization techniques have been described previously [32]. The probes were digoxigenin-labeled, after which we cloned their partial DNA fragments.

### mRNA preparation for the rescue experiment

Capped mRNAs of *gsk3α, gsk3β*, and *RFP *were synthesized according to the protocol of the manufacturer (Epicentre). The resultant mRNAs were diluted to 44 ng/μl with distilled water. Approximately 2.3 nl was injected into one-cell stage embryos.

### Detection of apoptotic cell death

The apoptosis assay was performed using The DeadEnd™ Colorimetric TUNEL System (Promega) and has been described previously [33].

## Authors' contributions

HCL designed and performed all the experiments, analysis the data, and prepared the manuscript. JNT and WCC carried out the Western blot analysis; KYL, PYL and WYT took care of fish handling including morpholino injections. CCC and CKS performed the two-photon fluorescence microscope imaging; HJT was a P.I. of this project. All authors read and approved the final manuscript.

## Supplementary Material

Additional file 1 Morphological phenotypes of zebrafish embryos derived from fertilized eggs injected with standard control morpholino. The standard negative control-MO has no target, no significant biological activity, and are commonly used in many studies (Hultman et al., 2007; Besser et al., 2007; Nixon et al., 2005). After 2 and 6 ng of this control MO were injected, no any defects were observed at 24 hpf. The morphology and development of heart appeared normally. Click here for file

Additional file 2 Similar cardiac defects in the *axin1 *and the *gsk3β *morphants. Axin1-MO or *gsk3β*-MO were microinjcetd to And observed under dissecting microscope by bright filed (A, C) or fluorescence (B, D). Incomplete looping of the heart tube was also observed in *axin1 *mutant heart. Click here for file

Additional file 3 Arrested pectoral fin bud induction in *gsk3β *morphants. At 72 hpf, wild-type pectoral fins elongate (A), but *gsk3β *morphants have still not developed fin buds (arrows; D). Whole mount in situ hybridization with *shh *and *dlx2 *staining reveal that the developed of fin bud were affected in *gsk3β *morphants. At 36 hpf, wild-type embryos continue *shh *(B) and *dlx2 *(C) expression in the developing bud mesenchyme, but in *gsk3β *morphants, the *shh *and *dlx2 *expression is greatly decreased. Click here for file

## References

[B1] Woodgett JR (1990). Molecular cloning and expression of glycogen synthase kinase-3/factor A. EMBO J.

[B2] Plyte SE, Hughes K, Nikolakaki E, Pulverer BJ, Woodgett JR (1992). Glycogen synthase kinase-3: functions in oncogenesis and development. Biochim Biophys Acta.

[B3] Hardt SE, Sadoshima J (2002). Glycogen synthase kinase-3beta: a novel regulator of cardiac hypertrophy and development. Circ Res.

[B4] van Amerongen R, Berns A (2005). Re-evaluating the role of Frat in Wnt-signal transduction. Cell Cycle.

[B5] Woodgett JR (1991). cDNA cloning and properties of glycogen synthase kinase-3. Methods Enzymol.

[B6] Woodgett JR (2001). Judging a protein by more than its name: GSK-3. Sci STKE.

[B7] Ali A, Hoeflich KP, Woodgett JR (2001). Glycogen synthase kinase-3: properties, functions, and regulation. Chem Rev.

[B8] Tsai JN, Lee CH, Jeng H, Chi WK, Chang WC (2000). Differential expression of glycogen synthase kinase 3 genes during zebrafish embryogenesis. Mech Dev.

[B9] Hoeflich KP, Luo J, Rubie EA, Tsao MS, Jin O, Woodgett JR (2000). Requirement for glycogen synthase kinase-3beta in cell survival and NF-kappaB activation. Nature.

[B10] Liang MH, Chuang DM (2007). Regulation and function of glycogen synthase kinase-3 isoforms in neuronal survival. J Biol Chem.

[B11] Sutherland C, Cohen P (1994). The alpha-isoform of glycogen synthase kinase-3 from rabbit skeletal muscle is inactivated by p70 S6 kinase or MAP kinase-activated protein kinase-1 in vitro. FEBS Lett.

[B12] Sutherland C, Leighton IA, Cohen P (1993). Inactivation of glycogen synthase kinase-3 beta by phosphorylation: new kinase connections in insulin and growth-factor signalling. Biochem J.

[B13] Goode N, Hughes K, Woodgett JR, Parker PJ (1992). Differential regulation of glycogen synthase kinase-3 beta by protein kinase C isotypes. J Biol Chem.

[B14] Nikoulina SE, Ciaraldi TP, Mudaliar S, Mohideen P, Carter L, Henry RR (2000). Potential role of glycogen synthase kinase-3 in skeletal muscle insulin resistance of type 2 diabetes. Diabetes.

[B15] Wojtaszewski JF, Nielsen P, Kiens B, Richter EA (2001). Regulation of glycogen synthase kinase-3 in human skeletal muscle: effects of food intake and bicycle exercise. Diabetes.

[B16] Markuns JF, Wojtaszewski JF, Goodyear LJ (1999). Insulin and exercise decrease glycogen synthase kinase-3 activity by different mechanisms in rat skeletal muscle. J Biol Chem.

[B17] Cohen P, Goedert M (2004). GSK3 inhibitors: development and therapeutic potential. Nat Rev Drug Discov.

[B18] Meijer L, Flajolet M, Greengard P (2004). Pharmacological inhibitors of glycogen synthase kinase 3. Trends Pharmacol Sci.

[B19] Bhat RV, Budd Haeberlein SL, Avila J (2004). Glycogen synthase kinase 3: a drug target for CNS therapies. J Neurochem.

[B20] Antos CL, McKinsey TA, Frey N, Kutschke W, McAnally J, Shelton JM, Richardson JA, Hill JA, Olson EN (2002). Activated glycogen synthase-3beta suppresses cardiac hypertrophy in vivo. Proc Natl Acad Sci U S A.

[B21] Badorff C, Ruetten H, Mueller S, Stahmer M, Gehring D, Jung F, Ihling C, Zeiher AM, Dimmeler S (2003). Fas receptor signaling inhibits glycogen synthase kinase 3s and induces cardiac hypertrophy following pressure overload. J Clin Invest.

[B22] Haq S, Choukroun G, Kang ZB, Ranu H, Matsui T, Rosenzweig A, Molkentin JD, Alessandrini A, Woodgett J, Hajjar R, Michael A, Force T (2000). Glycogen Synthase Kinase-3s Is a Negative Regulator of Cardiomyocyte Hypertrophy. J Cell Biol.

[B23] Michael A, Haq S, Chen X, Hsich E, Cui L, Walters B, Shao Z, Bhattacharya K, Kilter H, Huggins G, Andreucci M, Periasamy M, Solomon RN, Liao R, Patten R, Molkentin JD, Force T (2004). Glycogen synthase kinase-3beta regulates growth, calcium homeostasis, and diastolic function in the heart. J Biol Chem.

[B24] Foley AC, Mercola M (2005). Heart induction by Wnt antagonists depends on the homeodomain transcription factor Hex. Genes Dev.

[B25] Harris JA, Francannet C, Pradat P, Robert E (2003). The epidemiology of cardiovascular defects, part 2: a study based on data from three large registries of congenital malformations. Pediatr Cardiol.

[B26] Shader RI, Greenblatt DJ (1990). Lithium and the newborn heart. J Clin Psychopharmacol.

[B27] Zierler S (1985). Related Maternal drugs and congenital heart disease. Obstet Gynecol.

[B28] Tsai TH, Lin CY, Tsai HJ, Chen SY, Tai SP, Lin KH, Sun CK (2006). Biomolecular imaging based on far-red fluorescent protein with a high two-photon excitation action cross section. Opt Lett.

[B29] Huang CJ, Tu CT, Hsiao CD, Hsieh FJ, Tsai HJ (2003). Germ-line transmission of a myocardium-specific GFP transgene reveals critical regulatory elements in the cardiac myosin light chain 2 promoter of zebrafish. Dev Dyn.

[B30] Kimmel CB, Ballard WW, Kimmel SR, Ullmann B, Schilling TF (1995). Stages of embryonic development of the zebrafish. Dev Dyn.

[B31] Link V, Shevchenko A, Heisenberg CP (2006). Proteomics of early zebrafish embryos. BMC Dev Biol.

[B32] Lee HC, Huang HY, Lin CY, Chen YH, Tsai HJ (2006). Foxd3 mediates zebrafish myf5 expression during early somitogenesis. Dev Biol.

[B33] Lin CY, Yung RF, Lee HC, Chen WT, Chen YH, Tsai HJ (2006). Myogenic regulatory factors Myf5 and Myod function distinctly during craniofacial myogenesis of zebrafish. Dev Biol.

[B34] Stainier DY (2001). Zebrafish genetics and vertebrate heart formation. Nat Rev Genet.

[B35] Chen JN, van Eeden FJ, Warren KS, Chin A, Nusslein-Volhard C, Haffter P, Fishman MC (1997). Left-right pattern of cardiac BMP4 may drive asymmetry of the heart in zebrafish. Development.

[B36] Ramsdell AF (2005). Left-right asymmetry and congenital cardiac defects: getting to the heart of the matter in vertebrate left-right axis determination. Dev Biol.

[B37] Hurlstone AF, Haramis AP, Wienholds E, Begthel H, Korving J, Van Eeden F, Cuppen E, Zivkovic D, Plasterk RH, Clevers H (2003). The Wnt/beta-catenin pathway regulates cardiac valve formation. Nature.

[B38] Phiel CJ, Wilson CA, Lee VM, Klein PS (2003). GSK-3alpha regulates production of Alzheimer's disease amyloid-beta peptides. Nature.

[B39] Viatour P, Dejardin E, Warnier M, Lair F, Claudio E, Bureau F, Marine JC, Merville MP, Maurer U, Green D, Piette J, Siebenlist U, Bours V, Chariot A (2004). GSK3-mediated BCL-3 phosphorylation modulates its degradation and its oncogenicity. Mol Cell.

[B40] King TD, Bijur GN, Jope RS (2001). Caspase-3 activation induced by inhibition of mitochondrial complex I is facilitated by glycogen synthase kinase-3beta and attenuated by lithium. Brain Res.

[B41] Song L, De Sarno P, Jope RS (2002). Central role of glycogen synthase kinase-3beta in endoplasmic reticulum stress-induced caspase-3 activation. J Biol Chem.

[B42] Stoica BA, Movsesyan VA, Lea PM, Faden AI (2003). Ceramide-induced neuronal apoptosis is associated with dephosphorylation of Akt, BAD, FKHR, GSK-3beta, and induction of the mitochondrial-dependent intrinsic caspase pathway. Mol Cell Neurosci.

[B43] Klein PS, Melton DA (1996). A molecular mechanism for the effect of lithium on development. Proc Natl Acad Sci U S A.

[B44] Eldar-Finkelman H (2002). Glycogen synthase kinase 3: an emerging therapeutic target. Trends Mol Med.

[B45] Martinez A, Castro A, Dorronsoro I, Alonso M (2002). Glycogen synthase kinase 3 (GSK-3) inhibitors as new promising drugs for diabetes, neurodegeneration, cancer, and inflammation. Med Res Rev.

[B46] Sommer L (2005). Checkpoints of melanocyte stem cell development. Sci STKE.

[B47] Willert K, Brown JD, Danenbergn E, Duncan AW, Weissman IL, Reya T, Yates JR, Nusse R (2003). Wnt proteins are lipid-modified and can act as stem cell growth factors. Nature.

[B48] Chen S, Guttridge DC, You Z, Zhang Z, Fribley A, Mayo MW, Kitajewski J, Wang CY (2001). Wnt-1 signaling inhibits apoptosis by activating beta-catenin/T cell factor-mediated transcription. J Cell Biol.

[B49] You L, He B, Uematsu K, Xu Z, Mazieres J, Lee A, McCormick F, Jablons DM (2004). Inhibition of Wnt-1 signaling induces apoptosis in beta-catenin-deficient mesothelioma cells. Cancer Res.

[B50] Liang J, Slingerland JM (2003). Multiple roles of the PI3K/PKB (Akt) pathway in cell cycle progression. Cell Cycle.

[B51] Brar BK, Stephanou A, Knight R, Latchman DS (2002). Activation of protein kinase B/Akt by urocortin is essential for its ability to protect cardiac cells against hypoxia/reoxygenation-induced cell death. J Mol Cell Cardiol.

[B52] Germack R, Griffin M, Dickenson JM (2004). Activation of protein kinase B by adenosine A1 and A3 receptors in newborn rat cardiomyocytes. J Mol Cell Cardiol.

[B53] Barrow JR, Thomas KR, Boussadia-Zahui O, Moore R, Kemler R, Capecchi MR, McMahon AP (2003). Ectodermal Wnt3/beta-catenin signaling is required for the establishment and maintenance of the apical ectodermal ridge. Genes Dev.

[B54] Hill TP, Taketo MM, Birchmeier W, Hartmann C (2006). Multiple roles of mesenchymal beta-catenin during murine limb patterning. Development.

[B55] Garrity DM, Childs S, Fishman MC (2001). The heartstrings mutation in zebrafish causes heart/fin Tbx5 deficiency syndrome. Development.

[B56] Li QY, Newbury-Ecob RA, Terrett JA, Wilson DI, Curtis AR, Yi CH, Gebuhr T, Bullen PJ, Robson SC, Strachan T, Bonnet D, Lyonnet S, Young ID, Raeburn JA, Buckler AJ, Law DJ, Brook JD (1997). Holt-Oram syndrome is caused by mutations in TBX5, a member of the Brachyury (T) gene family. Nat Genet.

[B57] Liberatore CM, Searcy-Schrick RD, Yutzey KE (2000). Ventricular expression of tbx5 inhibits normal heart chamber development. Dev Biol.

[B58] Bruneau BG, Nemer G, Schmitt JP, Charron F, Robitaille L, Caron S, Conner DA, Gessler M, Nemer M, Seidman CE, Seidman JG (2001). A murine model of Holt-Oram syndrome defines roles of the T-box transcription factor Tbx5 in cardiogenesis and disease. Cell.

[B59] Takeuchi JK, Koshiba-Takeuchi K, Suzuki T, Kamimura M, Ogura K, Ogura T (2003). Tbx5 and Tbx4 trigger limb initiation through activation of the Wnt/Fgf signaling cascade. Development.

[B60] Atilla-Gokcumen GE, Williams DS, Bregman H, Pagano N, Meggers E (2006). Organometallic compounds with biological activity: a very selective and highly potent cellular inhibitor for glycogen synthase kinase 3. Chembiochem.

[B61] Dominguez I, Itoh K, Sokol SY (1995). Role of glycogen synthase kinase 3b as a negative regulator of dorsoventral axis formation in *Xenopus *embryos. Proc Natl Acad Sci USA.

[B62] Pierce SB, Kimelman D (1995). Regulation of Spemann organizer formation by the intracellular kinase Xgsk-3. Development.

