# Supplementary material for: Glycogen synthase kinase 3α and 3β have distinct functions during cardiogenesis of zebrafish embryo
Source: BMC Dev Biol. 2007 Aug 3;7:93. doi: 10.1186/1471-213X-7-93 (PMC1988812; doi:10.1186/1471-213X-7-93)
Supplement: Additional file 1 — Morphological phenotypes of zebrafish embryos derived from fertilized eggs injected with standard control morpholino. The standard negative control-MO has no target, no significant biological activity, and are commonly used in many studies (Hultman et al., 2007; Besser et al., 2007; Nixon et al., 2005). After 2 and 6 ng of this control MO were injected, no any defects were observed at 24 hpf. The morphology and development of heart appeared normally. [file 1471-213X-7-93-S1.doc]

Additional file 1

Morphological phenotypes of zebrafish embryos derived from fertilized eggs injected with standard control morpholino

| Injected materials | Concentration | Number of embryos surviving among number of injected eggs | Number of wild-type phenotype embryos among number of embryos surviving | Number of abnormal phenotype embryos among number of embryos surviving | Number of normal heart position embryos among number of wild-type phenotype embryos | Number of abnormal heart position embryos among number of wild-type phenotype embryos |
| --- | --- | --- | --- | --- | --- | --- |
| Injection dye | － | 38/39 | 37/38 (97.4%) | 1/38 (2.6%) | 35 (94.6%) | 2 (5.4%) |
| Control MO | 2 ng | 105/108 | 105/105 (100%) | 0/105 (0%) | 101 (96.2%) | 4 (3.8%) |
| Control MO | 6 ng | 112/125 | 110/112 (98.2%) | 2/112 (1.8%) | 103 (93.6%) | 7 (6.4%) |
| Fertilized eggs were injected at the 1-cell stage, and then observed the morphology at 24 hpf; the heart positions were analysis at 48 hpf. | | | | | | |
