# Supplementary material for: Glycogen synthase kinase 3α and 3β have distinct functions during cardiogenesis of zebrafish embryo
Source: BMC Dev Biol. 2007 Aug 3;7:93. doi: 10.1186/1471-213X-7-93 (PMC1988812; doi:10.1186/1471-213X-7-93)
Supplement: Additional file 2 — Similar cardiac defects in the axin1 and the gsk3β morphants. Axin1-MO or gsk3β-MO were microinjcetd to And observed under dissecting microscope by bright filed (A, C) or fluorescence (B, D). Incomplete looping of the heart tube was also observed in axin1 mutant heart. [file 1471-213X-7-93-S2.doc]

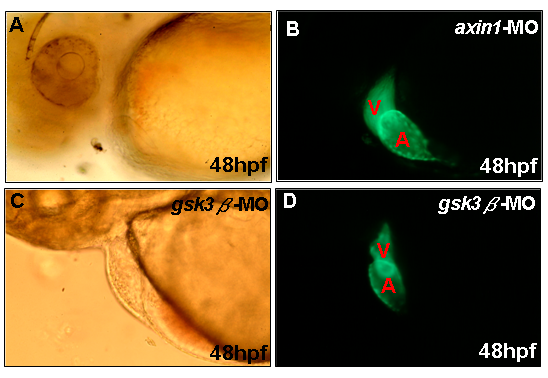


Additional file 2

Similar cardiac defects in the *axin1* and the *gsk3β* morphants. Axin1 MO or gsk3*β*MO were microinjcetd to And observed under dissecting microscope by bright filed (A, C) or fluorescence (B, D). Incomplete looping of the heart tube was also observed in *axin1* mutant heart.
