# Supplementary material for: Glycogen synthase kinase 3α and 3β have distinct functions during cardiogenesis of zebrafish embryo
Source: BMC Dev Biol. 2007 Aug 3;7:93. doi: 10.1186/1471-213X-7-93 (PMC1988812; doi:10.1186/1471-213X-7-93)
Supplement: Additional file 3 — Arrested pectoral fin bud induction in gsk3β morphants. At 72 hpf, wild-type pectoral fins elongate (A), but gsk3β morphants have still not developed fin buds (arrows; D). Whole mount in situ hybridization with shh and dlx2 staining reveal that the developed of fin bud were affected in gsk3β morphants. At 36 hpf, wild-type embryos continue shh (B) and dlx2 (C) expression in the developing bud mesenchyme, but in gsk3β morphants, the shh and dlx2 expression is greatly decreased. [file 1471-213X-7-93-S3.doc]

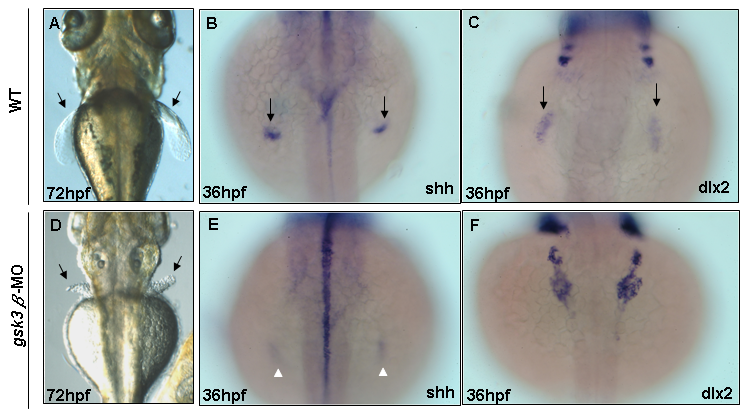


Additional file 3

Arrested pectoral fin bud induction in *gsk3β* **morphants**. At 72 hpf, wild-type pectoral fins elongate (A), but *gsk3β* **morphants** have still not developed fin buds (arrows; D). Whole mount in situ hybridization with *shh* and *dlx2* staining reveal that the developed of fin bud were affected in *gsk3β* **morphants**. At 36 hpf, wild-type embryos continue *shh* (B) and *dlx2* (C) expression in the developing bud mesenchyme, but in *gsk3β* **morphants, the *shh* and *dlx2*** expression is greatly decreased.
